# Approaching ramp lesions from the different world of posterior knee compartment: A review of evidence with a proposal of a new classification and treatment

**DOI:** 10.1002/jeo2.70018

**Published:** 2024-10-04

**Authors:** Sohrab Keyhani, Alireza Mirahmadi, Arash Maleki, Fardis Vosoughi, Rene Verdonk, Robert F. LaPrade, Philippe Landreau, Mohammad Movahedinia

**Affiliations:** ^1^ Bone Joint and Related Tissues Research Center, Akhtar Orthopedic Hospital Shahid Beheshti University of Medical Sciences Tehran Iran; ^2^ Department of Orthopedic and Trauma Surgery Tehran University of Medical Sciences Tehran Iran; ^3^ Department of Orthopedics and Traumatology Gent University Ghent Belgium; ^4^ Department of Orthopedic Surgery University of Minnesota Minneapolis Minnesota USA; ^5^ Consultant Orthopaedic Surgeon Knee, Shoulder and Sports Surgery Orthocure & Mediclinic Dubai UAE

**Keywords:** anterior cruciate ligament, arthroscopy, classification, meniscus, posterior knee compartment, ramp lesion, treatment

## Abstract

Ramp lesions (RLs) are peripheral lesions that occur in the posterior part of the medial meniscus or where it attaches to the joint capsule. The classification of the medial meniscus RLs has been the focus of numerous studies and publications. This review provides an overview of RL's current classification and treatment options in anterior cruciate ligament deficient knees. The study also aims to present a more practical classification system for RLs to assist in treatment decision‐making. For the first time, we also presented a new surgical treatment for incomplete inferior and double‐complete RL based on the posterior knee arthroscopy that provides direct access to the posterior meniscal borders, enabling effective treatment and stronger biomechanical repair.

**Level of Evidence:**

Level V.

**Abbreviations**
ACL: anterior cruciate ligament
LEAP: lateral extra‐articular procedure
MCL: medial collateral ligament
MRI: magnetic resonance imaging
RLs: ramp lesions

## INTRODUCTION

Ramp lesions (RLs) are peripheral lesions that occur in the posterior part of the medial meniscus or where they attach to the joint capsule, which involves peripheral meniscocapsular attachment of posterior horn of medial meniscus and meniscotibial ligament (MTL) disruption [7, 21]. These injuries are often associated with anterior cruciate ligament (ACL) tears [21]. The prevalence of RLs in knees with ACL deficiencies ranges from 9.3% to 40% [8, 13]. However, there is no agreed‐upon definition for RLs that may explain the variation in prevalence rates across studies [8].

**Figure 1:**
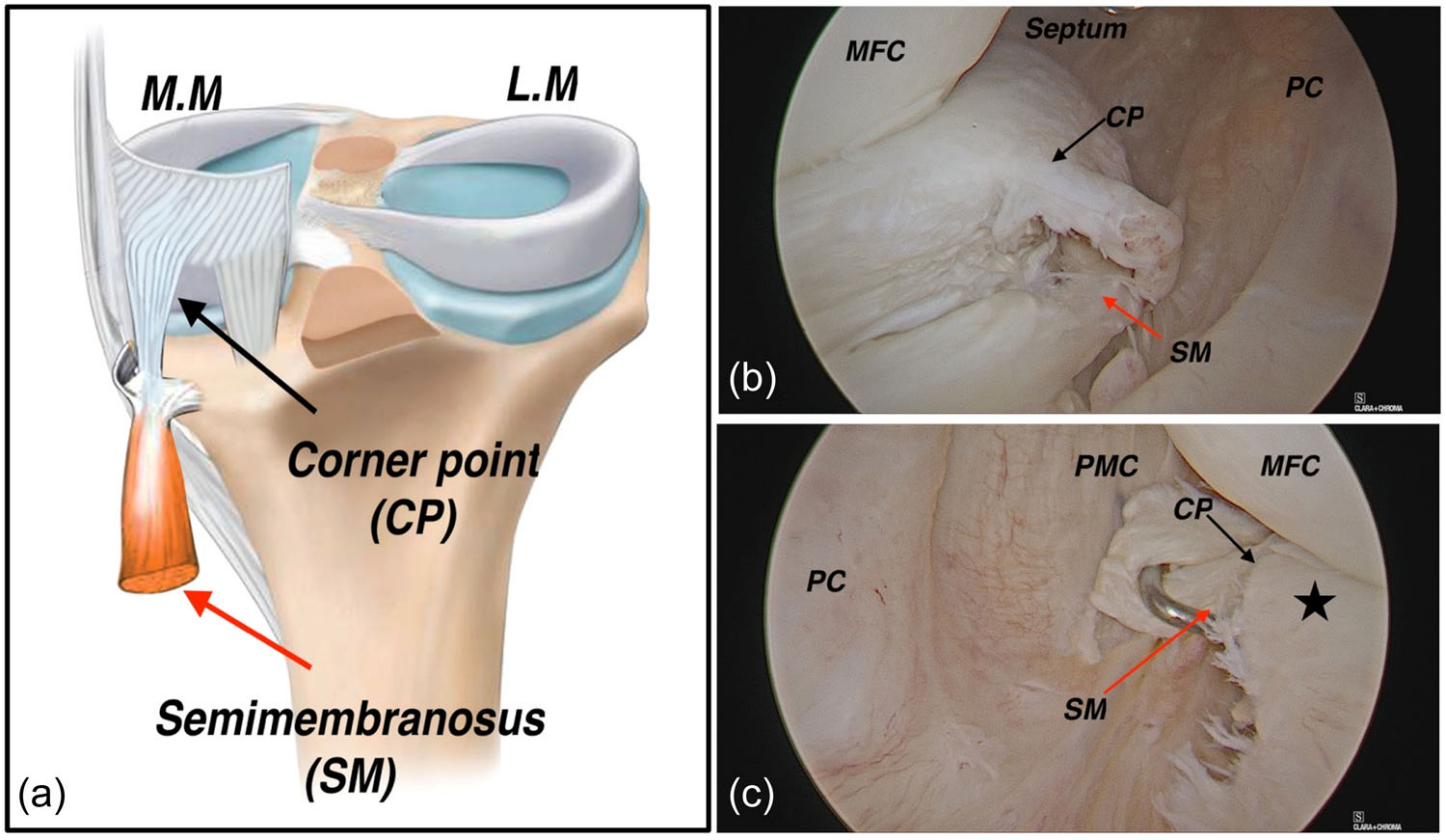
Anatomy of the medial meniscal corner point. (a) Schematic view, (b) right knee arthroscopic posteromedial view, (c) arthroscopic posterolateral transseptal view. Asterisk: medial meniscus. CP, corner point; LM, Lateral meniscus; MFC, medial femoral condyle; MM, medial meniscus; PC, posterior capsule; PMC, posteromedial capsule; SM, semimembranosus.

The posterior horn of the medial meniscus, its connection to the joint capsule and the meniscofemoral ligament all play important roles in treating tears in this area and determining the appropriate treatment approach [31]. However, this area's anatomy has been evaluated, and no consensus exists on how RLs should be defined [20]. The corner point, located between the posterior and middle thirds of the medial meniscus, is considered the starting point of RLs when the semimembranosus muscle contracts [34] (Figure 1). Biomechanical studies have shown that cutting the posterior meniscocapsular attachment site can result in increased laxity in external movements [7, 17, 26, 46]. These findings highlight the importance of RLs in terms of their potential impact on knee stability.

Detecting RLs can be challenging because they are often missed during preoperative magnetic resonance imaging (MRI) scans [18]. The accuracy of using MRI to detect meniscus pathologies has been evaluated [6, 14], and the sensitivity of MRI in detecting RLs ranges from 48% to 86% [10, 41]. Therefore, maintaining a level of suspicion is crucial, and additional imaging alterations, such as tibial bone bruising, may assist in diagnosing RLs [21, 22, 52]. It is worth noting that despite arthroscopy being the standard for diagnosing RLs, these lesions can still be easily missed through standard anterior portals [10].

Regarding RLs risk factors, Kunze et al. evaluate risk factors related to the RLs in a systematic review. They reported significant associations between the presence of RLs and male sex, age <30 years, posteromedial tibial oedema on MRI, concomitant lateral meniscal tears, complete ACL tears and injury chronicity. Also, they showed that contact injury and revision ACL reconstruction were not significantly associated with the presence of RLs [36].

Numerous studies and publications have focused on classifying RLs in the medial meniscus. Different authors have proposed classification systems to categorize these lesions based on their characteristics and the involvement of specific structures in the ramp area. The classification systems proposed by Thaunat et al. and Greif et al. have been widely used in subsequent studies and have provided a framework for understanding and categorizing RLs [8, 50]. These classification systems have helped in the diagnosis and treatment planning of patients with RLs. The question raised here is whether all types of RLs are included in these classifications and whether these classifications are sufficient for properly treating RLs.

**Figure 2:**
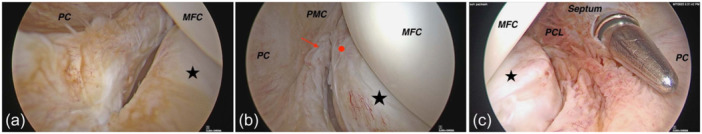
Comparative views of a right knee with a ramp lesion (RL): (a) Gillquist view, (b) posterolateral transseptal view, (c) posteromedial view. Asterisk: medial meniscus. MFC, medial femoral condy; PC, posterior capsule; PCL, posterior cruciate ligament; PMC, posteromedial capsule.

The management of RLs has been a topic of debate, with various treatment options and techniques suggested. Treatment choice depends on factors such as the stability of the lesion, associated injuries and the patient's symptoms. Controversy exists regarding the identification strategies and surgical interventions for RLs, with some advocating for repair and others questioning the need for treatment [15, 20]. Due to their location, these lesions may not be easily visible during anterior arthroscopy and may require additional posterolateral transseptal or posteromedial portals for proper confirmation [28] (Figure 2). Keyhani et al. hypothesized that bucket handle medial meniscus tear starts as an RL, and they emphasized routine posterior knee exams during ACL reconstruction [28].

**Figure 3:**
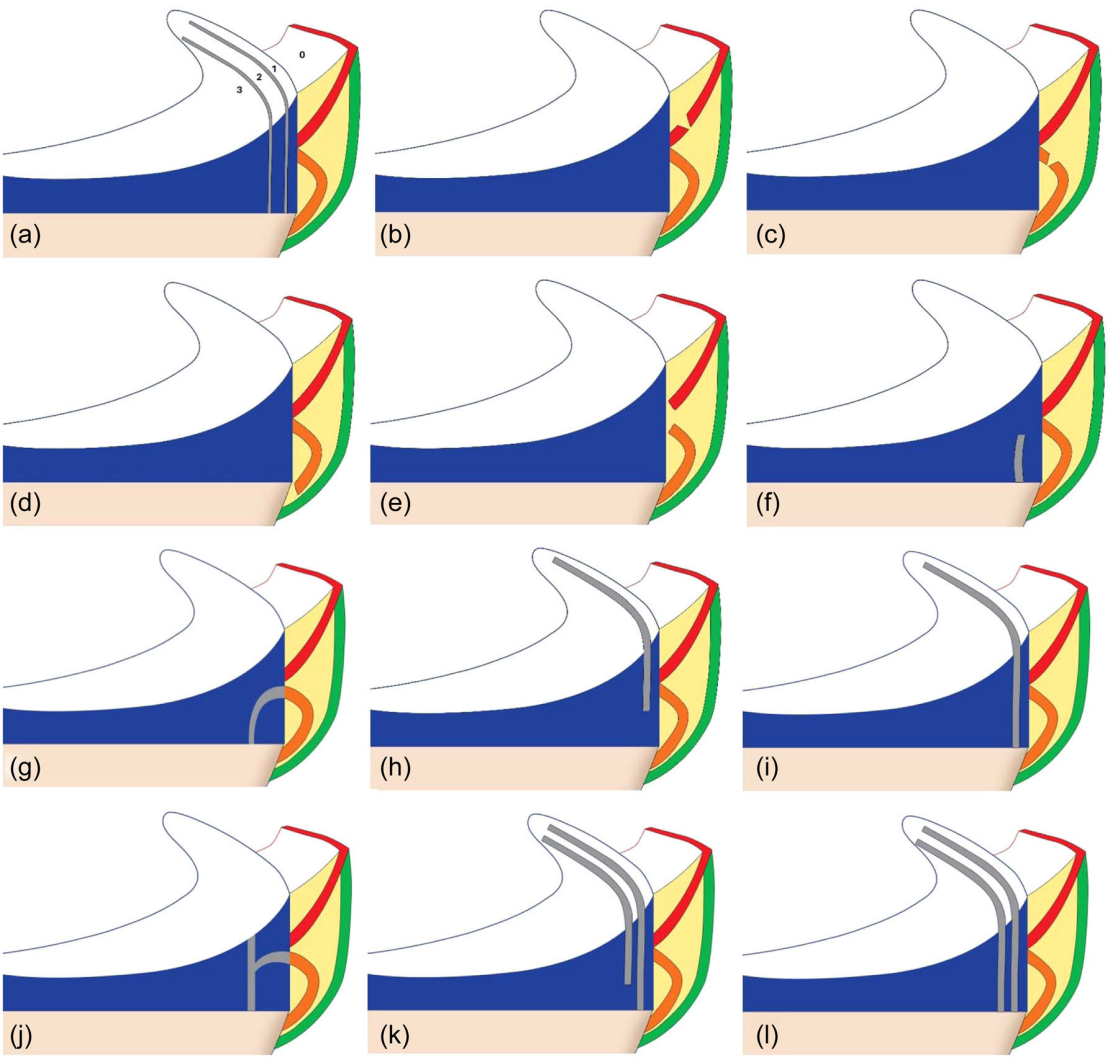
Schematic classification of medial meniscal ramp lesions (RLs). Red line: Meniscofemoral ligament, orange line: meniscotibial ligament, green line: Posterior capsule. (a) Four zones of the medial meniscus. 0: Meniscocapsular junction. 1: outer third 2: middle third 3: inner third, (b) meniscofemoral tear, (c) meniscotibial tear from meniscus, (d) meniscotibial from tibial, (e) both meniscofemoral and meniscotibial ligaments tears from the meniscus, (f) incomplete inferior, (g) incomplete inferior with posterior expansion, (h) incomplete superior, (i) complete tear, (j) complete with posterior expansion, (k) double tear but one incomplete, (l) double complete tears.

**Figure 4:**
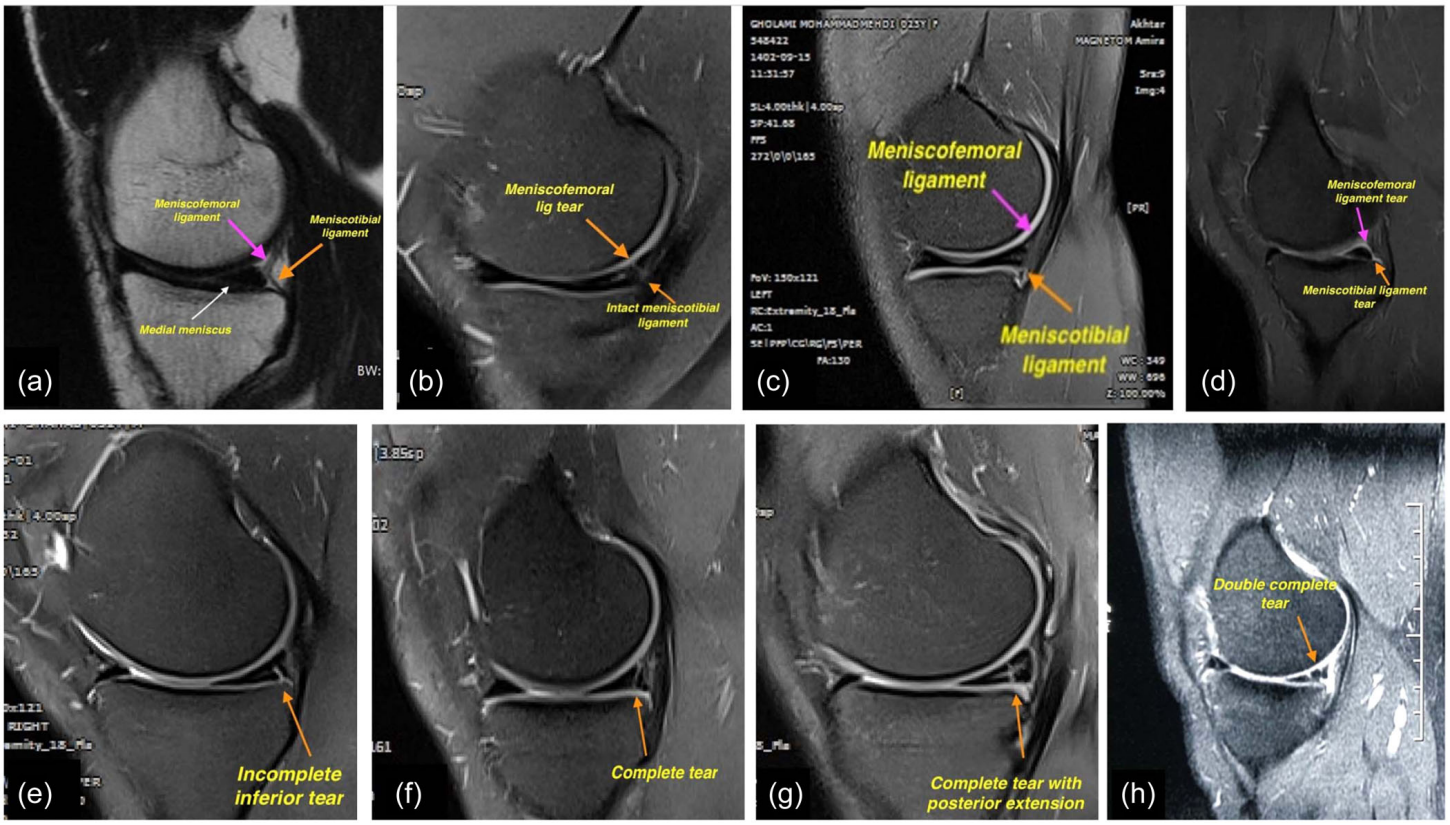
Magnetic resonance imaging (MRI) images of medial meniscal ramp lesions (RLs). (a) Normal meniscus, (b) meniscofemoral tear, (c) meniscotibial from meniscus, (d) both meniscofemoral and meniscotibial from meniscus, (e) incomplete inferior, (f) complete single, (g) complete single with posterior extension, (h) double complete.

**Figure 5:**
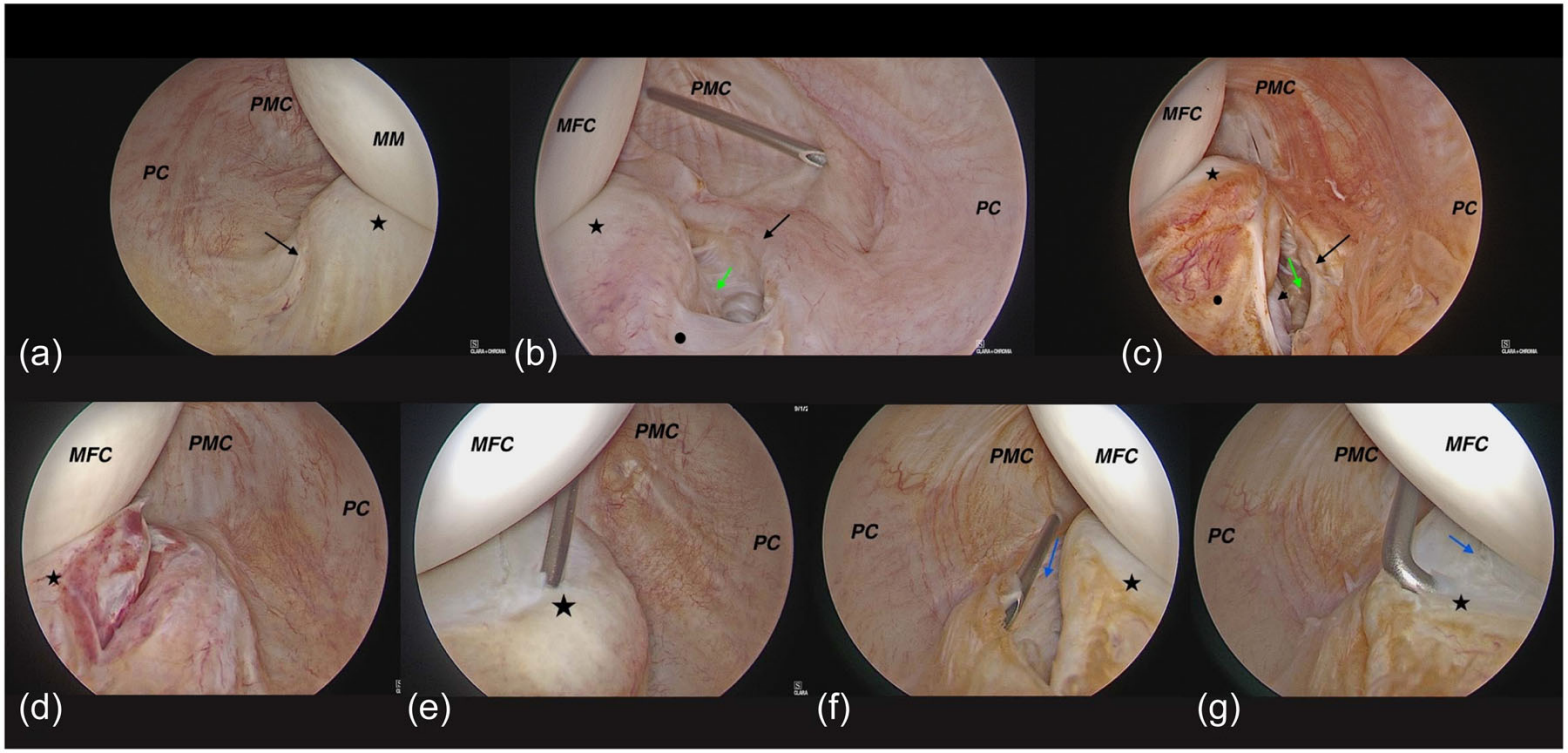
Arthroscopic poeterolateral transseptal view images of medial meniscal ramp lesions (RLs). (a) Normal meniscus of RT knee from a posterolateral trans septal view, (b) meniscofemoral tear of the left knee, (c) both meniscofemoral and meniscotibial from meniscus in the left knee, (d) Complete single (red zone) in the left knee, (e) complete single (red‐white zone) in the Left knee, (f, g) double incomplete in the right knee. MFC, medial femoral condyle; MM and Asterisk: medial meniscus. PC, posterior capsule; PMC, posteromedial capsule, Dot: Meniscotibial attachment, black arrow: meniscofemoral ligament, green arrow: meniscotibial ligament, blue arrow: Incomplete tear, arrowhead: Tibial plateau.

The success of arthroscopic repair for RLs has been demonstrated in several studies. For example, a study by Beaufils et al. found that repair of RLs showed high success rates in terms of recovery time, functional outcome and cartilage protection [9]. Another study by Thaunat et al. evaluated the results of arthroscopic repair of RLs and found a low failure rate [50]. In addition to arthroscopic repair, other strategies for RLs include nonoperative treatment and meniscal abrasion/trephination. However, nonoperative treatment may only be considered for stable lesions of the lateral meniscus during ACL reconstruction [9]. Meniscal abrasion/trephination has been used as a treatment option for stable RLs, with some authors suggesting that stable RLs can heal spontaneously if the lesion is stable after simple abrasion and trephination in a well‐stabilized knee [51]. However, the criteria for determining stable or unstable tears and the adequacy of examination with a probe remain unanswered. Also, the question here is whether it is possible to completely abrade and remove the fibrotic tissue from the anterior and whether there is a difference between what we see from the anterior and the posterior.

This review article aims to provide an overview of the current classification and treatment options of RLs in ACL‐deficient knees. The study also aims to present a more practical classification system of RLs. We also presented a new surgical treatment for incomplete inferior and double‐complete RLs for the first time.

## CURRENT CLASSIFICATIONS

In 2016, Thaunant et al. [49] classified RLs into different types based on tear pattern (partial or full‐thickness tear) and associated MTL disruption. This classification system included five types: meniscocapsular lesion, partial superior lesion, partial inferior lesion or hidden type, complete tear and double tear. In 2020, Greif et al. expanded on this classification system and proposed a more detailed classification with seven categories [24]. Their classification system considered the involvement of different structures in the ramp area. The study included seven categories: type 1 referred to a tear in the meniscocapsular ligament; type 2 was a tear in the peripheral meniscal horn; type 3‐A was a partial tear in the inferior peripheral posterior horn of the meniscus; type 3‐B indicated a tear in the MTL; type 4‐A was a complete tear in the peripheral posterior horn of the meniscus; type 4‐B referred to a complete tear at the junction between the meniscus and its capsule and, finally, type 5 represented a double tear in the peripheral posterior horn of the meniscus.

### Current treatments

The treatment for RLs during ACL reconstruction is still a matter of debate. Some studies suggest that nonsurgical management may be reasonable, while others suggest that repairing the lesion would be the most effective approach [11]. The healing status of RLs can affect postoperative knee stability after ACL reconstruction [26].

Differentiating between the size and stability of RLs is essential because small RLs that withstand arthroscopic evaluation with a probe may be managed nonoperatively [43]. Several factors come into play when deciding whether to continue nonoperative management or to perform a meniscectomy or repair for RLs, such as the stability of the lesion and the patient's age. It has been observed that stable RLs can sometimes heal without requiring repair, and unstable lesions are more likely to benefit from repair [12, 26]. In a study by Liu et al., 88% of stabled RLs treated with abrasion and trephination (as the nonsurgery group) had healed on follow‐up MRI scans after ACL reconstruction, and it was not statistically different compared to 95% healing rate in the surgery group [38].

However, if the lesion is unstable or there are accompanying ACL injuries, it is recommended to opt for repair of the RL [11, 26]. It should be noted that performing meniscectomy, which is typically subtotal or total, has been associated with lower outcomes for ACL reconstruction and an increased risk of future arthritis after ACL rupture. Also, several studies have evaluated the rate of secondary meniscectomy after failure of RL repair at the time of ACL reconstruction (ACLR). Sonnery‐Cottet et al. reported an 11% rate of meniscectomy at a follow‐up time of 45.6 months after inside‐out meniscus ramp repair. They showed that ACLR with anterolateral ligament reconstruction had a >2‐fold reduction in the risk of secondary meniscectomy [48]. Another study found the percentage of meniscectomy after inside‐out ramp repair to be 2% within 33.6 months [19]. Pioger et al. reported a 7.7% secondary meniscectomy rate at a mean final follow‐up of 72.4 months after ramp repair was performed using a posteromedial portal suture hook. They showed that ACLR with the lateral extra‐articular procedure (LEAP) could decrease the secondary meniscectomy by up to 50% [44]. These findings suggest that meniscectomy rates following ramp repair are relatively low.

Arthroscopic repair has been proposed as the treatment of choice for RLs, mainly during ACL reconstruction [1, 45, 47]. The goal of ramp repair is to improve knee biomechanics and promote meniscal healing. Several studies have reported favourable outcomes with the arthroscopic repair of RLs [26, 27]. Thaunat et al. conducted a study to evaluate the survival and risk factors associated with arthroscopic RL repair during ACL reconstruction. They found that the type of lesion (location, longitudinal extension, partial‐ or full‐thickness tear) and the suture technique used were associated with the failure of RL repair [50]. However, RLs can be missed in the “classic” anterior or anterolateral arthroscopic approach, highlighting the importance of preoperative suspicion and thorough evaluation of the posteromedial knee compartment [28, 35].

Using posterior portals to repair RLs has gained attention in recent years. This approach offers several advantages, including better visualization and improved diagnosis, the ability to perform a complete abrasion and remove fibrotic tissue from the meniscal borders without the need for medial collateral ligament (MCL) needling (which is necessary for the anterior approach), anatomical reduction of the meniscal borders, and closure of the lesion [28]. MCL needling in knee surgeries has been used, but some iatrogenic injuries and subsequent adverse outcomes have been reported [5, 16]. The use of posterior portals for RL repair has also been supported by studies that have evaluated the prevalence and risk factors of RLs in patients undergoing ACL reconstruction. Sonnery‐Cottet et al. found that different tear types are associated with different failure rates, and therefore, performing ramp repairs specifically through a posteromedial portal can help differentiate RLs from other types of meniscal lesions [47].

In terms of surgical techniques, various methods have been described for repairing RLs. All‐inside and inside‐out suture techniques have been commonly used, with favourable outcomes reported [23]. All‐inside suture repair, using a suture hook through a posteromedial portal, has shown a high rate of meniscal healing [26]. The FasT‐Fix technique, which involves inserting a FasT‐Fix needle obliquely close to the tibial plateau and using self‐sliding knots for secure fixation, is effective in repairing peripheral attachment lesions of the posterior horn of the medial meniscus [37]. Several studies have reported high healing rates for RLs after suture repair using a suture hook through the posteromedial portal [26]. For example, Ahn et al. conducted second‐look arthroscopy and reported complete healing of posterior horn tears in all knees that underwent all‐inside suture repair through the posteromedial portal [26]. This suggests that using posterior portals can lead to successful repair outcomes for RLs, possibly due to the complete abrasion, anatomical reduction and secure fixation achieved by this method.

### Novel classification and treatment

Previous classifications attempted to evaluate RLs comprehensively, yet they only offered an indirect perspective through anterior arthroscopy without considering the posterior view. Consequently, the posterior compartment was overlooked in knee arthroscopies. The new classifications underscores the significance of incorporating and instructing on posterior knee arthroscopy for addressing RLs. It is essential to recognize that procedures such as debridement, abrasion of meniscus edges and anatomical reduction cannot be effectively carried out during anterior knee arthroscopy, which is critical for treating and recovering RLs [30, 32, 34]. With this detailed classification, surgeons can make informed decisions on whether to utilize anterior or posterior knee arthroscopy for optimal meniscus treatment. This classification is anticipated to enhance the decision‐making process, thus improving patient outcomes and serving as a reference for future research.

Prior research commonly indicated the necessity of addressing visible knee pathologies. However, relying solely on probe assessment for RL stability assessment is insufficient and could potentially mislead surgeons, emphasizing the need for additional biomechanical investigations. We assert that the impact of sports activities on RL motion and stability differs significantly from evaluating stability with a probe during knee arthroscopy. Consequently, a lesion deemed stable through anterior arthroscopy evaluation may yield a false negative result, leading to patient complications. Due to the low sensitivity of the current method of evaluating RLS stability during anterior arthroscopy, it may be better to approach and repair any stable or unstable RLs associated with ACL tears. However, the cost‐effectiveness of this decision should be evaluated in terms of the risk of overtreatment. Past studies have highlighted the detrimental consequences of overlooking and not treating RLs on the outcomes of ACL treatment [34], underscoring the motivation behind proposing this novel classification system and emphasizing the advantages of posterior knee arthroscopy. According to this classification, we have five major types and 14 subgroups (Tables 1 and 2).

**Table 1:** Our new classification for medial meniscal ramp lesions (RLs).

Classification	Definition	Schematic figure	Magnetic resonance imaging figure	Arthroscopic figure
Type 1				
A	Meniscofemoral tear	Figure 3b	Figure 4b	Figure 5b
B1	Meniscotibial from meniscus	Figure 3c		
B2	Meniscotibial from tibial	Figure 3d	Figure 4c	
C	Both meniscofemoral and Meniscotibial from meniscus	Figure 3e	Figure 4d	Figure 5c
Type 2				
A	Incomplete inferior	Figure 3f	Figure 4e	Figure 6
B	Incomplete inferior with posterior extension	Figure 3g		
Type 3				
A	Incomplete superior red zone	Figure 3h		
B	Incomplete superior red‐white zone			
Type 4				
A	Complete single	Figure 3i	Figure 4f	
A1	Red zone			Figure 5d
A2	Red‐white zone			Figure 5e
B	Complete single with posterior extension	Figure 3j	Figure 4g	
B1	Red zone			
B2	Red‐white zone			
Type 5				
A	Double incomplete	Figure 3k		Figure 5f,g
B	Double complete	Figure 3l	Figure 4h	Figure 7

**Figure 6:**
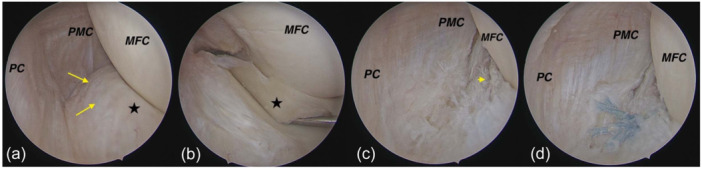
In type 2‐A, abnormal movement of the meniscus is observed. Posterolateral transseptal view of right knee. (a) The posterior view of the superior part of the right knee's meniscus shows no tear but a colour change (degeneration) and consistency of the meniscus (Yellow arrow). (b) abnormal meniscal movement during probe examination from the anterior. (c) A tear appeared after removal of superior meniscal tissue (arrowhead). (d) Final repair. Asterisk: medial meniscus. MFC, medial femoral; PC, posterior capsule; PMC, posteromedial capsule.

**Figure 7:**
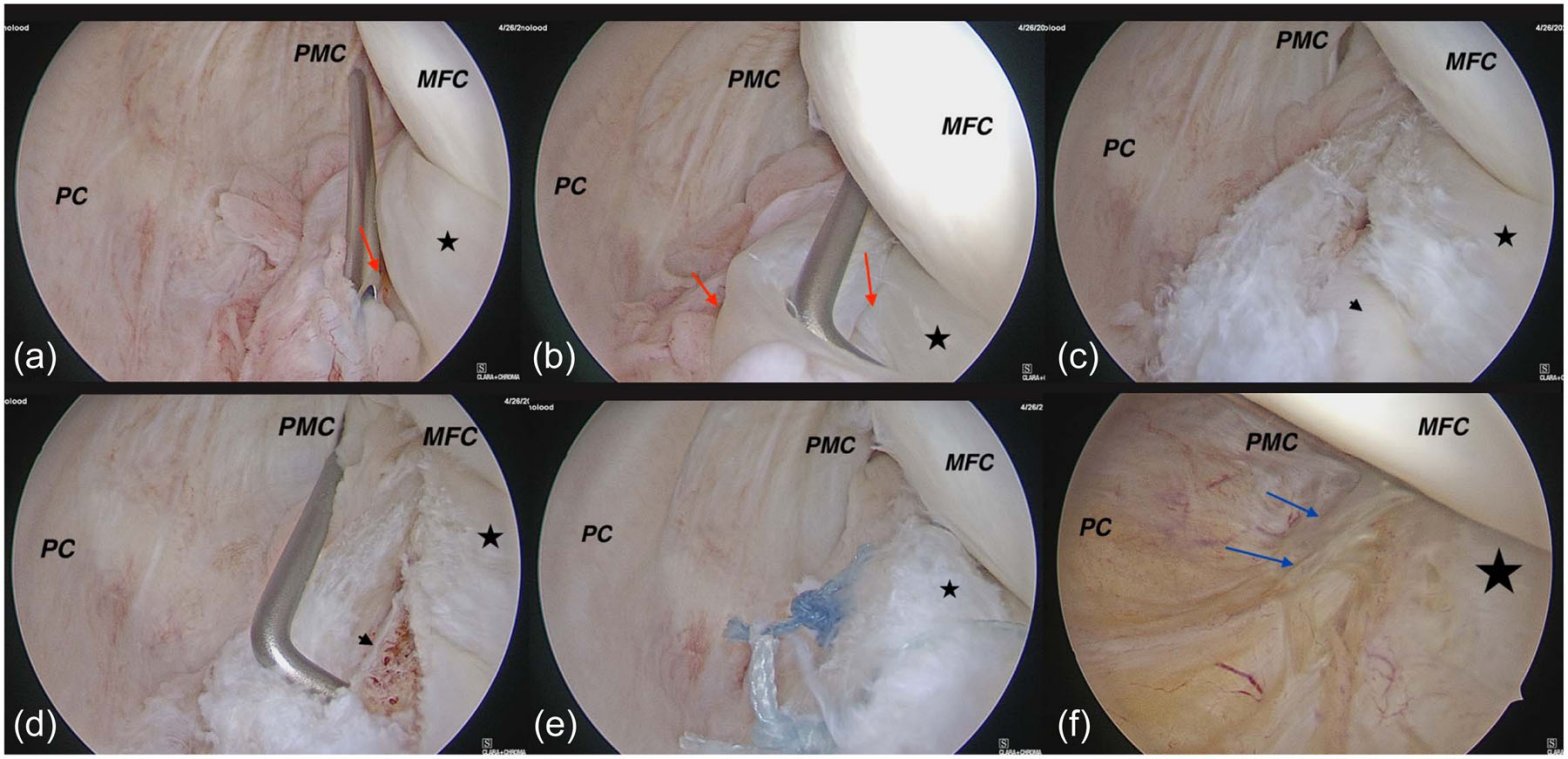
In the case of type 5‐B, when there is a double complete tear (red arrow), the avascular fragment is resected utilizing a shaver, and the tear is repaired as simple Ramp lesions (RLs) through a posterior arthroscopy. (a–b) Posterolateral transseptal view of right knee. Type 5‐B Ramp lesion (Double complete tear). (c) Avascular fragment is resected utilizing a shaver to become Ramp type 4‐A. (d) Abrasion chondroplasty of the tibial plateau promotes in situ clot formation. (e) Final repair. (f) In second look arthroscopy blue arrows shows complete healing. MFC, medial femoral condyle; PC, posterior capsule; PMC, posteromedial capsule. Asterisk: Medial meniscus, Arrowhead: Tibial plateau.

Repair can be done through posterior portals for types 1‐A, 1‐B1, and 1‐C. In type 1‐B2, if instability is visible during arthroscopy, fixation to the tibial plateau is performed. For hidden lesions (type 2‐A), if there is MRI evidence of a tear and abnormal movement of the meniscus with a probe, the surgeon must complete the tear with a shaver through posterior portals and then repair it through either an anterior or posterior approach (Figure 6). In type 3‐A, the tear is repaired through posterior portals, while in type 3‐B, repair can be done through an anterior approach. In type 4, similar to type 3, repair in the red zone is performed from the posterior, and in the red‐white zone, repair is done from the anterior. In type 5‐A, for a double incomplete tear, repair of the peripheral fragment and abrasion of the central tear is sufficient. In type 5‐B, for a double complete tear, the free avascular fragment is removed using a shaver, and the tear is repaired using a posterior approach (Figure 7). In all types, if the posteromedial part of the tibial plateau is visible, chondroplasty of this part may aid meniscal healing due to in‐situ clot formation (Figure 7d) (Table 2).

**Table 2:** New treatment suggestions for medial meniscal ramp lesions (RLs), based on our newly proposed classification.

Classification	Definition	Suggested treatment approach
Type 1		
A	Meniscofemoral tear	Posterior approach
B1	Meniscotibial from meniscus	Unstable lesions by posterior approach
B2	Meniscotibial from tibial	
C	Both meniscofemoral and Meniscotibial from meniscus	
Type 2		
A	Incomplete inferior	Stable lesion: Abrasion from posterior Unstable lesion: Complete the tear by posterior approach and repair via the anterior or posterior approach
B	Incomplete inferior with posterior extension	
Type 3		
A	Incomplete superior red zone	Stable lesion: Abrasion from posterior Unstable lesion: Abrasion from posterior and repair by anterior or posterior approach
B	Incomplete superior red‐white zone	Stable lesion: Abrasion from anteriorUnstable lesion: Abrasion and repair by anterior approach
Type 4		
A	Complete single	
A1	Red zone	Abrasion and repair by posterior approach
A2	Red‐white zone	Abrasion and repair by anterior approach
B	Complete single with posterior extension	
B1	Red zone	Abrasion and repair by posterior approach
B2	Red‐white zone	Abrasion and repair by anterior approach
Type 5		
A	Double incomplete	Abrasion and repair by posterior approach
B	Double complete	Abrasion, resection of the segmental fragment, and repair by posterior approach

## DISCUSSION

The new classification system emphasizes the importance of incorporating posterior knee arthroscopy in evaluating and treating RLs in knee arthroscopies. Unlike previous classifications that overlooked the posterior view [4], this approach allows for crucial procedures, such as meniscus abrasion and debridement, essential for RL healing. This classification aims to improve patient outcomes and guide future research by providing detailed treatment strategies based on posterior arthroscopy, offering a more comprehensive and effective approach to managing RLs. Management of RLs involves clinical evaluation, imaging studies, and arthroscopic techniques. Treatment should be based on the specific characteristics of the lesion and the patient's symptoms. Nonoperative treatment or meniscal abrasion/trephination may be considered for stable RLs. According to a recent systematic review [3], patients with untreated stable RLs had similar clinical outcomes compared to those without an RL. Therefore, the authors concluded that treating stable RLs during ACL reconstruction does not provide clinical benefit. Another study by Liu et al. compared the outcomes of ACL reconstruction with and without surgical repair of stable RLs [38]. Studies found that treating stable RLs with abrasion and trephination alone resulted in similar clinical outcomes compared to surgical repair [38, 40]. This suggests that not all RLs require surgical intervention and that conservative treatment options may be effective in certain cases.

However, arthroscopic repair is often recommended for unstable RLs or lesions associated with ACL tears [28, 50]. Sonnery‐Cottet et al. conducted a study to evaluate the incidence and risk factors for RLs in patients undergoing ACL reconstruction [47]. They found that the overall incidence of RLs in the study population was 23.9%, highlighting the prevalence of these lesions in ACL‐injured knees. The study also demonstrated that ramp repair performed during ACL reconstruction could improve knee biomechanics [47]. Current literature aligns with the necessity of ramp repair to restore knee biomechanics, especially in reducing anterior and rotational laxity in ACL‐deficient knees [2, 39, 42]. Leaving RLs unrepaired may also increase cartilage degeneration in the medial compartment of the knee [25].

Our proposed treatment strategies for each RL type, based on the posterior arthroscopic approach, are outlined in this article, accompanied by second‐look images. Several studies support the role of posterior knee arthroscopy in treating RLs. Keyhani et al. reported excellent clinical results after the repair of RLs using the posterolateral transseptal portal view [28]. They proposed abrasion chondroplasty of the posteromedial part of the tibial plateau to enhance meniscal healing, which is only accessible through a posterior approach [28, 34] Figure 7d. They also suggested that all bucket‐handle medial meniscus tears can be classified as RLs after reduction [29, 33]. It is important to note that RLs can be easily missed through standard anterior portals during arthroscopy [10]. Therefore, posterior knee arthroscopy provides direct access to the posterior meniscal borders, allowing for adequate abrasion, fibrous tissue removal, and stronger biomechanical repair using vertical mattress sutures [29]. This technique is particularly useful in unstable RLs, which may have significant biomechanical consequences and require surgical repair [23].

However, the key question that remains is whether relying on a probe for testing lesion stability is adequate, particularly in athletes and highly active individuals. For proponents of abrasion as a sufficient treatment, the concern revolves around the abrasion method of this part of the meniscus from the anterior. In our opinion, the location of the tear in the red or red‐white zone plays a crucial role in the decision‐making process for the repair technique and the success of the repair. Additionally, there is no consensus on the repair technique, especially for type III (incomplete inferior) and V Thannat double ramp tears. In type V tears, the avascular mid‐fragment may contribute to failure, similar to the role of a sequestered fragment in nonunion fractures.

We proposed a new classification for RLs to assist in treatment decision‐making and present our treatment approach, particularly for type V lesions. Based on this new classification, in the scenario of type 5‐A, where a double incomplete tear is present, it is deemed adequate to undertake repair of the peripheral fragment and engage in abrasion of the central tear. In the instance of type 5‐B, where a double complete tear is observed, the course of action entails the removal of the avascular fragment that is unattached, which is accomplished through the application of a shaver. Subsequently, the tear is addressed through the implementation of a posterior approach. We also presented a more practical approach to the hidden lesions (type 2‐A in the current classification); if there is observable evidence of a tear on an MRI scan, along with atypical movement of the meniscus during examination with a probe, the surgeon should fully address the tear using a shaver through posterior portals and subsequently, repair the tear using either an anterior or posterior approach.

Regarding the limitations of this study, we were deficient in terms of an adequately structured research strategy, inclusion and exclusion criteria, and the research process and analysis. These deficiencies have the potential to impede the effectiveness of this study. Furthermore, it is essential to note that this review was authored by a limited number of experts in a field, thus introducing the possibility of bias and potentially overlooking a comprehensive array of research pertaining to the subject matter. Consequently, it is suggested that any feasible potential associated with the novel classification and treatment methods presented in this study be evaluated in forthcoming randomized studies that compare various methodologies. Interestingly, a remarkable part of the available literature on this subject comes from only four groups (La Prade, Seil, Sonnery‐Cottet, and Keyhani). Therefore, external validation is necessary with studies from other teams. Further research is needed to determine the optimal treatment approach for RLs and to compare different repair techniques.

## CONCLUSION

In summary, posterior knee arthroscopy is crucial for a better diagnosis and treating RLs. Posterior knee arthroscopy provides direct access to the posterior meniscal borders, enabling effective treatment and stronger biomechanical repair. It allows for detecting and repairing these lesions, improving knee biomechanics and stability. While conservative treatment options may be suitable for stable RLs, surgical repair is recommended for unstable lesions. A new classification of RLs was also introduced in this study.

## AUTHOR CONTRIBUTIONS

All authors contributed to the idea development and study design. Sohrab Keyhani, Alireza Mirahmadi, and Mohammad Movahedinia performed the literature search and drafted the manuscript. All authors contributed to critically revising the work and commented on previous versions of the manuscript. All authors read and approved the final manuscript.

## CONFLICT OF INTEREST STATEMENT

The authors declare no conflict of interest.

## ETHICS STATEMENT

Not applicable.

## References

[1] Acosta, J. , Ravaei, S. , Brown, S.M. & Mulcahey, M.K. (2020) Examining techniques for treatment of medial meniscal ramp lesions during anterior cruciate ligament reconstruction: a systematic review. Arthroscopy: The Journal of Arthroscopic & Related Surgery, 36, 2921–2933. Available from: 10.1016/j.arthro.2020.05.041 32674943

[2] Ahn, J.H. , Bae, T.S. , Kang, K.‐S. , Kang, S.Y. & Lee, S.H. (2011) Longitudinal tear of the medial meniscus posterior horn in the anterior cruciate ligament–deficient knee significantly influences anterior stability. The American Journal of Sports Medicine, 39, 2187–2193. Available from: 10.1177/0363546511416597 21828365

[3] Alessio‐Mazzola, M. , Lovisolo, S. , Capello, A.G. , Zanirato, A. , Chiarlone, F. , Formica, M. et al. (2020) Management of ramp lesions of the knee: a systematic review of the literature. Musculoskeletal Surgery, 104, 125–133. Available from: 10.1007/s12306-019-00624-z 31595426

[4] Allende, F. , Berreta, R.S. , Allahabadi, S. , Mowers, C. , Russo, R. , Palco, M. et al. (2024) Meniscal ramp lesion classification systems: a systematic review. Knee Surgery, Sports Traumatology, Arthroscopy, 32, 1710–1724. Available from: 10.1002/ksa.12188 38666656

[5] Amiri, S. , Mirahmadi, A. , Parvandi, A. , Hosseini‐Monfared, P. , Minaei Noshahr, R. , Hoseini, S.M. et al. (2024) Management of Iatrogenic medial collateral ligament injury in primary total knee arthroplasty: a systematic review. The Archives of Bone and Joint Surgery, 12, 159–166. Available from: 10.22038/ABJS.2023.73563.3406 38577515 PMC10989723

[6] Amiri, S. , Mirahmadi, A. , Parvandi, A. , Moshfegh, M.Z. , Hashemi Abatari, S.P. , Farrokhi, M. et al. (2024) Excellent accuracy of magnetic resonance imaging for diagnosis of discoid meniscus tears: a systematic review and meta‐analysis. Journal of Experimental Orthopaedics, 11, e12051. Available from: 10.1002/jeo2.12051 38899047 PMC11185948

[7] Bae, B.S. , Yoo, S. & Lee, S.H. (2023) Ramp lesion in anterior cruciate ligament injury: a review of the anatomy, biomechanics, epidemiology, and diagnosis. Knee Surgery & Related Research, 35, 23. Available from: 10.1186/s43019-023-00197-z 37626385 PMC10464050

[8] Balazs, G.C. , Greditzer, H.G. , Wang, D. , Marom, N. , Potter, H.G. , Marx, R.G. et al. (2019) Ramp lesions of the medial meniscus in patients undergoing primary and revision ACL reconstruction: prevalence and risk factors. Orthopaedic Journal of Sports Medicine, 7, 2325967119843509. Available from: 10.1177/2325967119843509 31205962 PMC6537250

[9] Beaufils, P. & Pujol, N. (2017) Management of traumatic meniscal tear and degenerative meniscal lesions. Save the meniscus. Orthopaedics & Traumatology: Surgery & Research, 103, S237–S244. Available from: 10.1016/j.otsr.2017.08.003 28873348

[10] Bumberger, A. , Koller, U. , Hofbauer, M. , Tiefenboeck, T.M. , Hajdu, S. , Windhager, R. et al. (2020) Ramp lesions are frequently missed in ACL‐deficient knees and should be repaired in case of instability. Knee Surgery, Sports Traumatology, Arthroscopy, 28, 840–854. Available from: 10.1007/s00167-019-05521-3 PMC7035224 31076825

[11] Chahla, J. , Dean, C.S. , Moatshe, G. , Mitchell, J.J. , Cram, T.R. , Yacuzzi, C. et al. (2016) Meniscal ramp lesions: anatomy, incidence, diagnosis, and treatment. Orthopaedic Journal of Sports Medicine, 4, 2325967116657815. Available from: 10.1177/2325967116657815 27504467 PMC4963625

[12] Cristiani, R. , Mouton, C. , Stålman, A. & Seil, R. (2023) Meniscal ramp lesions: a lot is known, but a lot is also unknown. Knee Surgery, Sports Traumatology, Arthroscopy, 31, 2535–2539. Available from: 10.1007/s00167-022-07292-w 36544052

[13] Cristiani, R. , van de Bunt, F. , Kvist, J. & Stålman, A. (2023) High prevalence of meniscal ramp lesions in anterior cruciate ligament injuries. Knee Surgery, Sports Traumatology, Arthroscopy, 31, 316–324. Available from: 10.1007/s00167-022-07135-8 PMC9859899 36045182

[14] D'Ambrosi, R. , Di Maria, F. , Ursino, C. , Ursino, N. , Di Feo, F. , Formica, M. et al. (2024) Magnetic resonance imaging shows low sensitivity but good specificity in detecting ramp lesions in children and adolescents with ACL injury: a systematic review. Journal of ISAKOS, 9, 371–377. Available from: 10.1016/j.jisako.2023.12.005 38135056

[15] D'Ambrosi, R. , Meena, A. , Raj, A. , Giorgino, R. , Ursino, N. , Mangiavini, L. et al. (2023) Good results after treatment of RAMP lesions in association with ACL reconstruction: a systematic review. Knee Surgery, Sports Traumatology, Arthroscopy, 31, 358–371. Available from: 10.1007/s00167-022-07067-3 PMC9859864 35869982

[16] da Silva Campos, V.C. , Guerra Pinto, F. , Constantino, D. , Andrade, R. & Espregueira‐Mendes, J. (2021) Medial collateral ligament release during knee arthroscopy: key concepts. EFORT Open Reviews, 6, 669–675. Available from: 10.1302/2058-5241.6.200128 34532074 PMC8419794

[17] Deichsel, A. , Miets, H. , Peez, C. , Raschke, M.J. , Klimek, M. , Glasbrenner, J. et al. (2024) The effect of varying sizes of ramp lesions in the ACL‐deficient and reconstructed knee: a biomechanical robotic investigation. The American Journal of Sports Medicine, 52, 928–935. Available from: 10.1177/03635465231223686 38343294

[18] DePhillipo, N.N. , Cinque, M.E. , Chahla, J. , Geeslin, A.G. , Engebretsen, L. & LaPrade, R.F. (2017) Incidence and detection of meniscal ramp lesions on magnetic resonance imaging in patients with anterior cruciate ligament reconstruction. The American Journal of Sports Medicine, 45, 2233–2237. Available from: 10.1177/0363546517704426 28463534

[19] DePhillipo, N.N. , Dornan, G.J. , Dekker, T.J. , Aman, Z.S. , Engebretsen, L. & LaPrade, R.F. (2020) Clinical characteristics and outcomes after primary ACL reconstruction and meniscus ramp repair. Orthopaedic Journal of Sports Medicine, 8, 2325967120912427. Available from: 10.1177/2325967120912427. 32426400 PMC7218952

[20] DePhillipo, N.N. , Moatshe, G. , Chahla, J. , Aman, Z.S. , Storaci, H.W. , Morris, E.R. et al. (2019) Quantitative and qualitative assessment of the posterior medial meniscus anatomy: defining meniscal ramp lesions. The American Journal of Sports Medicine, 47, 372–378. Available from: 10.1177/0363546518814258 30525875

[21] Doral, M.N. , Bilge, O. , Huri, G. , Turhan, E. & Verdonk, R. (2018) Modern treatment of meniscal tears. EFORT Open Reviews, 3, 260–268. Available from: 10.1302/2058-5241.3.170067 29951265 PMC5994634

[22] Green, J.S. , Moran, J. , Marcel, A. , Joo, P.Y. , McLaughlin, W.M. , Manzi, J.E. et al. (2023) Posteromedial tibial plateau bone bruises are associated with medial meniscal ramp lesions in patients with concomitant anterior cruciate ligament ruptures: a systematic review & meta‐analysis. The Physician and Sportsmedicine, 51, 531–538. Available from: 10.1080/00913847.2022.2108350 35915996

[23] Green, J.S. , Yalcin, S. , Moran, J. , McLaughlin, W.M. & Medvecky, M.J. (2022) Dual posteromedial portal technique for surgical repair of an unstable medial meniscal ramp lesion. Video Journal of Sports Medicine, 2, 26350254221122583. Available from: 10.1177/26350254221122583 PMC11923765 40308322

[24] Greif, D.N. , Baraga, M.G. , Rizzo, M.G. , Mohile, N.V. , Silva, F.D. , Fox, T. et al. (2020) MRI appearance of the different meniscal ramp lesion types, with clinical and arthroscopic correlation. Skeletal Radiology, 49, 677–689. Available from: 10.1007/s00256-020-03381-4 31982971

[25] Guimaraes, J.B. , Schwaiger, B.J. , Gersing, A.S. , Neumann, J. , Facchetti, L. , Li, X. et al. (2021) Meniscal ramp lesions: frequency, natural history, and the effect on knee cartilage over 2 years in subjects with anterior cruciate ligament tears. Skeletal Radiology, 50, 551–558. Available from: 10.1007/s00256-020-03596-5 32901305 PMC7854891

[26] Hatayama, K. , Terauchi, M. , Saito, K. , Takase, R. & Higuchi, H. (2020) Healing status of meniscal ramp lesion affects anterior knee stability after ACL reconstruction. Orthopaedic Journal of Sports Medicine, 8, 2325967120917674. Available from: 10.1177/2325967120917674 32426412 PMC7222250

[27] Karaca, M.O. , Özbek, E.A. , Ertan, M.B. , Terzi, M.M. & Akmeşe, R. (2022) Short‐term outcomes after treatment of isolated hidden meniscal ramp lesions. Orthopaedic Journal of Sports Medicine, 10, 23259671221085977. Available from: 10.1177/23259671221085977 35386838 PMC8977712

[28] Keyhani, S. , Ahn, J.H. , Verdonk, R. , Soleymanha, M. & Abbasian, M. (2017) Arthroscopic all‐inside ramp lesion repair using the posterolateral transseptal portal view. Knee Surgery, Sports Traumatology, Arthroscopy, 25, 454–458. Available from: 10.1007/s00167-016-4410-9 28028568

[29] Keyhani, S. , Movahedinia, M. , LaPrade, R.F. , Qoreishy, M. & Vosoughi, F. (2023) Long‐term clinical results of using a posteromedial all‐inside and anteromedial inside‐out approach to repair unstable or irreducible bucket‐handle medial meniscal tears. Journal of Orthopaedics and Traumatology, 24, 12. Available from: 10.1186/s10195-023-00691-w 37024629 PMC10079791

[30] Keyhani, S. , Movahedinia, M. , Sherafat Vaziri, A. , Soleymanha, M. , Vosoughi, F. , Tahami, M. et al. (2023) Is posterior knee arthroscopy using posterior portals necessary for orthopedic surgeons? The latest evidence on applications and techniques. EFORT Open Reviews, 8, 189–198. Available from: 10.1530/EOR-22-0133 37097043 PMC10155121

[31] Keyhani, S. , Movahedinia, M. , Soleymanha, M. , Verdonk, R. , Kazemi, M. & Qoreishy, M. (2021) Repair of popliteomeniscal fascicles tear using a posterior transseptal portal fixes hypermobile lateral meniscus. Journal of Experimental Orthopaedics, 8, 93. Available from: 10.1186/s40634-021-00412-4 34676494 PMC8531177

[32] Keyhani, S. , Soleymanha, M. , Verdonk, R. , Amouzadeh, F. , Movahedinia, M. & Kazemi, S.M. (2022) Posterior knee arthroscopy facilitates the safe and effective all‐inside repair of locked bucket‐handle medial meniscal tear using a suture hook technique. Knee Surgery, Sports Traumatology, Arthroscopy, 30, 1311–1315. Available from: 10.1007/s00167-021-06576-x 33871661

[33] Keyhani, S. , Soleymanha, M. , Verdonk, R. , Amouzadeh, F. , Movahedinia, M. & Kazemi, S.M. (2022) Posterior knee arthroscopy facilitates the safe and effective all‐inside repair of locked bucket‐handle medial meniscal tear using a suture hook technique. Knee Surgery, Sports Traumatology, Arthroscopy, 30, 1311–1315. Available from: 10.1007/s00167-021-06576-x 33871661

[34] Keyhani, S. , Vaziri, A.S. , Vosoughi, F. , Verdonk, R. & Movahedinia, M. (2022) Overview of posterior knee arthroscopy in the medial meniscal repair: technical note. Journal of ISAKOS, 7, 33–38. Available from: 10.1016/j.jisako.2022.02.002 36178394

[35] Koo, B. , Lee, S.H. , Yun, S.J. & Song, J.G. (2020) Diagnostic performance of magnetic resonance imaging for detecting meniscal ramp lesions in patients with anterior cruciate ligament tears: a systematic review and meta‐analysis. The American Journal of Sports Medicine, 48, 2051–2059. Available from: 10.1177/0363546519880528 31684739

[36] Kunze, K.N. , Wright‐Chisem, J. , Polce, E.M. , DePhillipo, N.N. , LaPrade, R.F. & Chahla, J. (2021) Risk factors for ramp lesions of the medial meniscus: a systematic review and meta‐analysis. The American Journal of Sports Medicine, 49, 3749–3757. Available from: 10.1177/0363546520986817 33565883

[37] Li, W. , Chen, Z. , Song, B. , Yang, R. & Tan, W. (2015) The FasT‐Fix repair technique for ramp lesion of the medial meniscus. Knee Surgery & Related Research, 27, 56–60. Available from: 10.5792/ksrr.2015.27.1.56 25750895 PMC4349646

[38] Liu, X. , Zhang, H. , Feng, H. , Hong, L. , Wang, X. & Song, G. (2017) Is it necessary to repair stable ramp lesions of the medial meniscus during anterior cruciate ligament reconstruction? a prospective randomized controlled trial. The American Journal of Sports Medicine, 45, 1004–1011. Available from: 10.1177/0363546516682493 28060534

[39] Marin, F. , Soto, J. , Barahona, M. & Negrin, R. (2023) Searching for the best treatment for ramp lesions: a systematic review and network meta‐analysis. Cureus, 15, e41651. Available from: 10.7759/cureus.41651 37435014 PMC10332486

[40] Moreira, J. , Almeida, M. , Lunet, N. & Gutierres, M. (2020) Ramp lesions: a systematic review of MRI diagnostic accuracy and treatment efficacy. Journal of Experimental Orthopaedics, 7, 71. Available from: 10.1186/s40634-020-00287-x 32978704 PMC7519018

[41] Moteshakereh, S.M. , Zarei, H. , Nosratpour, M. , Zaker Moshfegh, M. , Shirvani, P. , Mirahmadi, A. et al. (2024) Evaluating the diagnostic performance of MRI for identification of meniscal ramp lesions in ACL‐deficient knees: a systematic review and meta‐analysis. Journal of Bone and Joint Surgery, 106, 1117–1127. Available from: 10.2106/jbjs.23.00501 38595146

[42] Mouton, C. , Magosch, A. , Pape, D. , Hoffmann, A. , Nührenbörger, C. & Seil, R. (2020) Ramp lesions of the medial meniscus are associated with a higher grade of dynamic rotatory laxity in ACL‐injured patients in comparison to patients with an isolated injury. Knee Surgery, Sports Traumatology, Arthroscopy, 28, 1023–1028. Available from: 10.1007/s00167-019-05579-z 31250053

[43] Naendrup, J.‐H. , Pfeiffer, T.R. , Chan, C. , Nagai, K. , Novaretti, J.V. , Sheean, A.J. et al. (2019) Effect of meniscal ramp lesion repair on knee kinematics, bony contact forces, and in situ forces in the anterior cruciate ligament. The American Journal of Sports Medicine, 47, 3195–3202. Available from: 10.1177/0363546519872964 31560563

[44] Pioger, C. , Ayata, M. , Pettinari, F. , Ali, A.A. , Alayane, A. , Campos, J.P. et al. (2024) Secondary meniscectomy rates and risk factors for failed repair of ramp lesions performed at the time of primary ACL reconstruction: an analysis of 1037 patients from the SANTI study group. The American Journal of Sports Medicine, 52, 1944–1951. Available from: 10.1177/03635465241253841 38853744

[45] Pizza, N. , Urda, L.L. , Sanchez, F.S. , Ibañez, M. , Zaffagnini, S. , Perelli, S. et al. (2024) How to repair medial meniscal ramp lesions: a systematic review of surgical techniques. Journal of Experimental Orthopaedics, 11, e12037. Available from: 10.1002/jeo2.12037. 38887657 PMC11180972

[46] Severyns, M. , Zot, F. , Harika‐Germaneau, G. , Germaneau, A. , Herpe, G. , Naudin, M. et al. (2024) Extrusion and meniscal mobility evaluation in case of ramp lesion injury: a biomechanical feasibility study by 7T magnetic resonance imaging and digital volume correlation. Frontiers in Bioengineering and Biotechnology, 11, 1289290. Available from: 10.3389/fbioe.2023.1289290 38249805 PMC10796713

[47] Sonnery‐Cottet, B. , Praz, C. , Rosenstiel, N. , Blakeney, W.G. , Ouanezar, H. , Kandhari, V. et al. (2018) Epidemiological evaluation of meniscal ramp lesions in 3214 anterior cruciate ligament‐injured knees from the SANTI study group database: a risk factor analysis and study of secondary meniscectomy rates following 769 ramp repairs. The American Journal of Sports Medicine, 46, 3189–3197. Available from: 10.1177/0363546518800717 30307740

[48] Sonnery‐Cottet, B. , Praz, C. , Rosenstiel, N. , Blakeney, W.G. , Ouanezar, H. , Kandhari, V. et al. (2018) Epidemiological evaluation of meniscal ramp lesions in 3214 anterior cruciate ligament–injured knees from the SANTI study group database: a risk factor analysis and study of secondary meniscectomy rates following 769 ramp repairs. The American Journal of Sports Medicine, 46, 3189–3197. Available from: 10.1177/0363546518800717 30307740

[49] Thaunat, M. , Fayard, J.M. , Guimaraes, T.M. , Jan, N. , Murphy, C.G. & Sonnery‐Cottet, B. (2016) Classification and surgical repair of ramp lesions of the medial meniscus. Arthroscopy Techniques, 5, e871–e875. Available from: 10.1016/j.eats.2016.04.009 27709051 PMC5040630

[50] Thaunat, M. , Foissey, C. , Ingale, P. , Haidar, I. , Bauwens, P.H. , Penet, A. et al. (2022) Survival and risk factor analysis of arthroscopic ramp lesion repair during anterior cruciate ligament reconstruction. The American Journal of Sports Medicine, 50, 637–644. Available from: 10.1177/03635465211068524 35099318

[51] Tuphé, P. , Foissey, C. , Unal, P. , Vieira, T.D. , Chambat, P. , Fayard, J.‐M. et al. (2022) Long‐term natural history of unrepaired stable ramp lesions: a retrospective analysis of 28 patients with a minimum follow‐up of 20 years. The American Journal of Sports Medicine, 50, 3273–3279. Available from: 10.1177/03635465221120058 36074027

[52] Willinger, L. , Balendra, G. , Pai, V. , Lee, J. , Mitchell, A. , Jones, M. et al. (2022) Medial meniscal ramp lesions in ACL‐injured elite athletes are strongly associated with medial collateral ligament injuries and medial tibial bone bruising on MRI. Knee Surgery, Sports Traumatology, Arthroscopy, 30, 1502–1510. Available from: 10.1007/s00167-021-06671-z PMC9033723 34341846

